# Chronic Arsenical Poisoning

**Published:** 1900-12-01

**Authors:** 


### Chronic Arsenical Poisoning.

The sensation of the moment undoubtedly is the outbreak of peripheral neuritis which has occurred in Manchester and some other parts, and to Dr. Reynolds, of Manchester, is to be given the very great credit of having traced back the origin of this epidemic to chronic arsenical poisoning. From his account[1] it appears that women have been affected more than men, which is quite in accord with what we know about chronic arsenical poisoning, and that all the patients are drinkers of beer or porter. The symptoms complained of are generally pains in the feet and hands, with tingling and sensations of "pins and needles," pains in the limbs, loss of power in the legs and arms, and rashes on the body with much itching.

The appearance of these patients is so peculiar that their malady can be diagnosed at sight. The face is puffy, especially about the eyes; the eyes are suffused and watery; and the skin is frequently dark-coloured from pigmentation. The patients often walk into the room as if their feet were very sore, and some of them have typical "dropped foot," due to paralysis of the anterior tibial muscles. Almost all the cases show some pigmentation of the skin. In some this occurs in an extreme degree, but in others the pigment spots are isolated in patches on the trunk, the spots being about the size of a large pin-head or a hempseed, and the skin between being normal. Sometimes the pigmentation is seen to be concentrated round the hair-follicles, and wherever there has been pressure, as round the neck or the waist, or where garters have been worn, the staining is darker than elsewhere. The pigmentation much resembles that of Addison's disease. It does not occur in the palms of the hands or the soles of the feet. In many cases, again, there are itchy erytematous affections which after a time become covered with a scaly desquamation. Sometimes there is keratosis of the hands or feet, either uniform or in warty patches; sometimes there are blisters exactly like pemphigus; in some cases there is herpes zoster; while in a good many the signs presented are like erythromelalgia, the "red neuralgia" of Weir Mitchell. Various mental symptoms also are sometimes met with, such as the loss of memory of time and place which is so common in alcoholic paralysis; but taking the cases altogether, Dr. Reynolds says that the proportion suffering from mental confusion is less than in alcoholic paralytics. The nature of the paralysis, however, differs in but few points from that which is met with in ordinary alcoholic cases. In some instances the circulation is so enfeebled that there is œdema of the feet and legs. As regards the mucous membranes, there is running at the eyes, and sometimes in the early stages running at the nose and frontal headache, while the patients often suffer from bronchitis. In many cases the tongue is coated with a silvery "fur," gastric catarrh may be present, or there is stomatitis, and in many cases there is persistent vomiting.

What is clear is that the present outbreak is quite a new thing; that the disease is only met with in beer or porter drinkers; and that, although at first sight the symptoms are somewhat like those of alcoholic paralysis, they differ from them in the greater amount of sensory and vascular disturbance, and especially in the presence of peculiar skin lesions. Now there are but three conditions by which such symptoms of peripheral neuritis are liable to be caused — namely, beri-beri, alcohol, and arsenic. Neither beri-beri nor alcohol, however, will explain the condition of the skin which is found in this outbreak. Hence we are driven to arsenic as the cause, and all the more so from the fact that arsenic has actually been found in the beer.

Since the publication of Dr. Reynolds's article a considerable number of samples of beer have been analysed, with the result that arsenic has been found in them, and it has also been shown that in certain samples of what are commonly spoken of as "substitutes," namely, various preparations used by brewers in place of malt, arsenic is also present. Whence the arsenic arises in these preparations is being much disputed, but as their production often depends upon the action of acids, and particularly sulphuric acid, by aid of which in some cases starch is made to change more or less completely into sugar—or at least into a fermentable compound—while in other cases ordinary cane sugar is turned into "invert" sugar, which is found to be more suitable for brewing purposes; and as arsenic is one of the common impurities of the commoner kinds of sulphuric acid, it would seem most likely that it is to the acids used in preparing the "substitutes" that the mischief will be traced. Beer we have always had with us, therefore it is not the beer simple. Beri-beri does not pick out the beer drinkers, and its distribution is very different from what is found in these cases, although no doubt the *ensemble* presented by them is very like what is seen in this disease. "Substitutes," again, we have had with us for so long that we can hardly feel justified in condemning offhand, as some seem inclined to do, the use of such materials, from which undoubtedly, when used in moderation, very good beer can be brewed. The probabilities are that some ingenious gentleman has found out that he can, by using extraordinarily common acids, produce his substitutes at an extraordinarily cheap rate, and (possibly quite in ignorance of what he was doing) has put on the market some arsenicated material which a certain number of brewers have accepted without analysis. Hence all the trouble. But arsenic is a poison which it is very easy to discover, and there can be no doubt that if the sanitary authorities during the next few weeks exercise the powers they possess in regard to the analysis of foods and drinks, the whole thing can be quickly stamped out. After the present warning, any brewer who sells arsenicated beer will certainly do so at the peril of losing all his business.

Perhaps out of evil good may come. Perhaps for long enough arsenical poisoning in a minor and undiscoverable degree may have been the unsuspected cause of many ailments. Now, however, that we are on the scent of it such a form of sophistication ought never again to escape the attention of our analysts.

[1] Brit. Med. Jour., Nov. 24.

